# Position-specific differences in countermovement vertical jump force-time metrics in professional male basketball players

**DOI:** 10.3389/fspor.2023.1218234

**Published:** 2023-07-20

**Authors:** Dimitrije Cabarkapa, Nicolas M. Philipp, Damjana V. Cabarkapa, Andrew C. Fry

**Affiliations:** Department of Health, Sport and Exercise Sciences, Jayhawk Athletic Performance Laboratory—Wu Tsai Human Performance Alliance, University of Kansas, Lawrence, KS, United States

**Keywords:** testing, athletes, biomechanics, kinetic, kinematic, performance, monitoring

## Abstract

The countermovement vertical jump (CVJ) is one of the most commonly implemented non-invasive and time-efficient testing modalities for lower-body neuromuscular performance assessment. With more practitioners having access to portable force plates, the purpose of this study was to examine position-specific differences in CVJ force-time metrics within a cohort of elite professional male basketball athletes. Twenty-eight athletes competing in top-tier European basketball leagues volunteered to participate in the present study. Following familiarization with testing procedures and a standardized warm-up protocol, each athlete performed three maximal-effort CVJ on a uni-axial force plate system with hands on the hips during the entire movement. To minimize the possible influence of fatigue, each jump trial was separated by an approximately 15-s rest interval. The mean value across three jumps was used for performance analysis purposes. The findings of the present study reveal notable position-specific differences during the eccentric phase of the CVJ, with centers having greater braking impulse, mean force, and mean power when compared to guards. However, when normalized by body mass, the observed differences during the eccentric phase of the CVJ were nonexistent. On the other hand, no significant differences in absolute mean and peak force and power were detected during the concentric phase of the CVJ. Yet, when normalized by the player’s body mass, centers demonstrated inferior performance than guards for the same force-time metrics. Overall, these findings may help practitioners obtain a better insight into position-specific differences with regards to CVJ force-time characteristics as well as aid with individually tailored training regimen design.

## 1. Introduction

With exponential technological development over the last decade, the ability to quantify athletes’ neuromuscular performance has become more accessible to sports scientists and strength and conditioning practitioners working with a diverse spectrum of athletes. At the forefront of this evolution are force plates, which have become portable and migrated from laboratories to practice facilities and/or weight rooms. Some of the most commonly implemented assessments on the force plate include the countermovement vertical jump (CVJ) and drop jump variations, as well as squat and isometric tasks (1–4). More specifically, the CVJ aims to assess an athlete’s ability to utilize the stretch-shortening cycle as a phenomenon consisting of an eccentric phase followed by an isometric transitional period (i.e., amortization phase) that leads into a concentric action (5).

As a non-invasive testing modality, the CVJ has been implemented in return-to-play scenarios (6) to monitor athletes’ fatigue and readiness status (7), as well as to track longitudinal performance changes (8). From a more sport-specific standpoint, positional differences may be detected with regards to different physiological qualities observed within gameplay. For instance, when examining a cohort of professional basketball players, Koklu et al. (9) found that centers displayed inferior *T*-test and 10 and 30 m sprinting performance when compared to guards. Also, guards had significantly higher maximal oxygen uptake (VO_2max_) than centers (9). Alongside other position-specific differences in physical performance characteristics, Delextrat and Cohen (10) documented that guards tend to outperform both centers and forwards in suicide runs (i.e., faster times) and are capable of attaining greater single leg jump heights. Lastly, Pehar et al. (11) investigated position-specific and performance-level differences in various jump testing modalities (e.g., CVJ and standing broad jump) in professional male basketball players. The authors proposed that differences were observed with regard to jumping performance, in favor of the guards and forwards playing at the higher competitive levels (11). This type of descriptive information can be of great interest to sports scientists and strength and conditioning practitioners when performing similar types of physical performance testing with their athletes/teams, especially at the top-tier levels of sports competition.

While different variations of the jumping tasks were part of the test batteries highlighted in some of the previously mentioned research reports, the database pertaining to detailed lower-body neuromuscular performance is still somewhat scarce. With more practitioners having access to portable force plate systems, a descriptive analysis of position-specific differences with regard to CVJ force-time characteristics, especially within highly trained basketball athletes, could be a meaningful addition to the current body of literature. Thus, the purpose of the present study was to examine the position-specific differences in CVJ force-time metrics within a cohort of elite professional male basketball players.

## 2. Materials and methods

### 2.1. Participants

Twenty-eight elite professional male basketball players ($\bar{x}$  ± SD; age = 24.5 ± 5.2 years; height = 2.00 ± 0.08 m, body mass = 94.6 ± 8.6 kg) competing at top-tier European basketball leagues (e.g., Adriatic Basketball Association) volunteered to participate in the present study. All players were free of musculoskeletal injuries and were cleared for participation in team activities by their respective sports medicine staff. Testing procedures performed in this investigation were previously approved by the University’s Institutional Review Board, and all athletes signed an informed consent document.

### 2.2. Procedures

Upon arrival at the gym, athletes completed a standardized warm-up procedure administered by their respective strength and conditioning coach consisting of dynamic stretching exercises [e.g., forward/backpedal jog, forward/side lunges, high knees, butt kicks, carioca, and straight leg kicks (12)]. Following familiarization with testing procedures, each athlete stepped on a uni-axial force plate system (ForceDecks Max, VALD Performance, Brisbane, Australia) and performed three maximal-effort CVJ with hands on the hips during the entire movement. The force plate system, sampling at 1,000 Hz, was calibrated/zeroed between each participant. To minimize the possible influence of fatigue, each jump trial was separated by an approximately 15-s rest interval. The mean value across three jumps was used for performance analysis purposes. Verbal encouragement was provided to encourage participants to give maximal effort and focus on pushing the ground as forcefully as possible (13).

### 2.3. Variables

**Figure 1:**
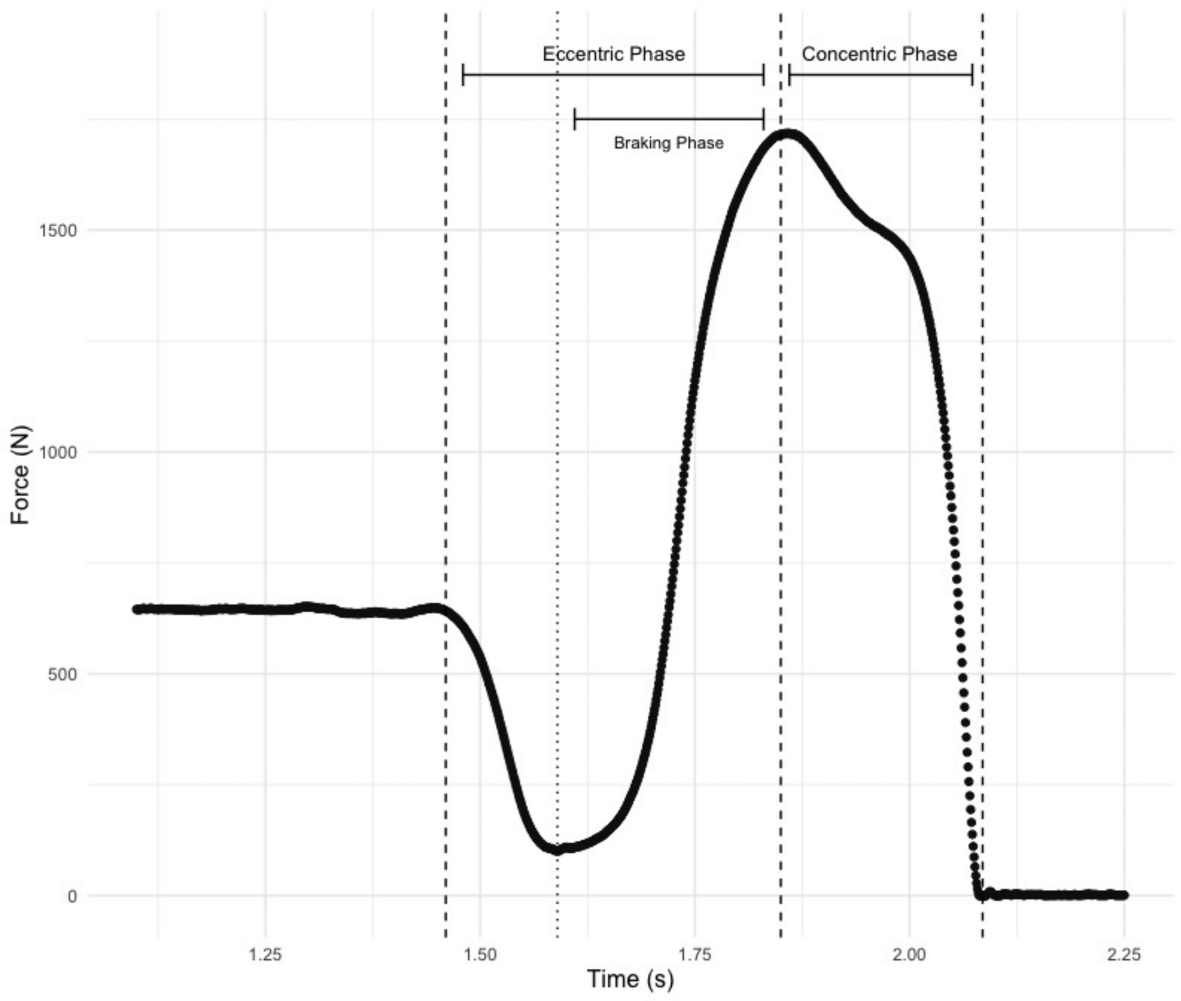
Graphical representation of countermovement vertical jump force-time curve.

The force-time metrics examined in the present study were selected based on previously published research reports (14–16). The following variables were examined during the eccentric phase of the CVJ: braking phase duration, braking impulse, braking impulse/body mass (BM), eccentric duration, peak velocity, mean force, mean force/BM, peak force, peak force/BM, mean power, mean power/BM, peak power, and peak power/BM. The following variables were examined during the concentric phase of the CVJ: impulse, impulse/BM, concentric duration, peak velocity, mean force, mean force/BM, peak force, peak force/BM, mean power, mean power/BM, peak power, and peak power/BM. In addition, contraction time, jump height, and modified reactive strength index (RSI-modified) were obtained. Jump height was calculated using the impulse-momentum relationship, while RSI-modified was calculated by dividing jump height by contraction time. The contraction time started when the athlete’s system mass was reduced by 20 N, which is termed the movement onset, and ended at take-off. Similarly, the take-off was defined as the timepoint at which vertical force dropped below a threshold of 20 N. In line with manufacturer recommendations, the eccentric phase was defined as the phase containing negative center of mass velocity. The braking phase was defined as a subphase of the eccentric phase, starting at minimum force until the end of the eccentric phase. Impulse within each respective sub-phase was calculated as the area under the force-time curve. A sample force-time curve with sub-phase definitions is presented in Figure 1. Additionally, a detailed description of CVJ force-time metrics can be found in the VALD user manual (https://valdperformance.com/forcedecks/) and previous research reports (15, 16).

### 2.4. Statistical analysis

Descriptive statistics, means and standard deviations ($\bar{x}$ ± SD), were calculated for each force-time metric. Shapiro-Wilk’s test corroborated that the assumption of normality was not violated for any of the dependent variables examined in the present study. A one-way analysis of variance (ANOVA) with Bonferroni post-hoc adjustments was used to examine statistically significant position-specific differences in force-time metrics between guards (*n* = 15; age = 23.3 ± 5.3 years; height = 2.03 ± 0.04 m, body mass = 98.6 ± 4.4 kg), forwards (*n* = 8; age = 24.4 ± 4.9 years; height = 1.94 ± 0.05 m, body mass = 89.0 ± 6.7 kg), and centers (*n* = 5; age = 27.0 ± 5.0 years; height = 2.08 ± 0.04 m, body mass = 105.2 ± 4.3 kg). Due to the within-group small sample size (*n* < 20), Hedge’s *g* was used to calculate the measure of effect size [i.e., *g *= 0.2 is a small effect, *g *= 0.5 is a moderate effect, and *g *> 0.8 is a large effect (17)]. Statistical significance was set *a priori* to *p *< 0.05. All statistical analyses were completed with SPSS (Version 26.0; IBM Corp., Armonk, NY, USA).

## 3. Results

During the eccentric phase of the CVJ, position-specific differences in force-time metrics were observed for braking impulse (*F*_[2,25] _= 6.003, *p *= 0.007), mean force (*F*_[2,25] _= 24.232, *p *< 0.001), and mean power (*F*_[2,25] _= 10.060, *p *< 0.001). Centers had notably greater braking impulse when compared to forwards (*p *= 0.040, *g *= 1.278) and guards (*p *= 0.006, *g *= 1.239). Mean force was significantly lower for forwards when compared to centers (*p *= 0.002, *g *= 2.165), and for guards when compared to forwards (*p *= 0.013, *g *= 1.419) and centers (*p *< 0.001, *g *= 3.420). Moreover, mean power was not significantly different between forwards and guards, but considerably greater for centers when compared to forwards (*p* = 0.023, *g* = 1.904) and guards (*p *< 0.001, *g *= 2.304). On the other hand, no statistically significant differences were found for eccentric phase duration (*F*_[2,25] _= 0.071, *p *= 0.932), braking phase duration (*F*_[2,25] _= 0.313, *p *= 0.734), braking impulse/BM (*F*_[2,25] _= 1.035, *p *= 0.370), peak velocity (*F*_[2,25] _= 0.696, *p *= 0.508), mean force/BM (*F*_[2,25] _= 0.522, *p* = 0.599), peak force (*F*_[2,25] _= 1.461, *p *= 0.251), peak force/BM (*F*_[2,25] _= 0.892, *p *= 0.422), mean power/BM (*F*_[2,25] _= 1.108, *p *= 0.346), peak power (*F*_[2,25] _= 1.354, *p *= 0.277), and peak power/BM (*F*_[2,25] _= 0.065, *p *= 0.937).

During the concentric phase of the CVJ, statistically significant differences were found between playing positions in concentric duration (*F*_[2,25] _= 4.025, *p *= 0.031), impulse (*F*_[2,25] _= 7.869, *p *= 0.002), mean force/BM (*F*_[2,25] _= 5.225, *p *= 0.012), peak force/BM (*F*_[2,25] _= 3.573, *p *= 0.043), mean power/BM (*F*_[2,25] _= 5.713, *p *= 0.009), and peak power/BM (*F*_[2,25] _= 5.983, *p *= 0.008) force-time metrics. Centers demonstrated greater concentric phase duration when compared to guards (*p *= 0.027, *g *= 1.419). Impulse was notably lower for guards when compared to centers (*p *= 0.006, *g *= 1.611) and forwards (*p *= 0.025, *g *= 1.238), although no difference was observed between centers and forwards. Guards had considerably greater mean force/BM when compared to centers (*p *= 0.010, *g *= 1.573). Peak force/BM was greater for guards when compared to centers (*p *= 0.042, *g *= 1.388). While no difference was observed between forwards and guards, mean power/BM was lower for centers when compared to forwards (*p *= 0.042, *g *= 2.294) and guards (*p *= 0.008, *g *= 1.610), Also, peak power/BM was significantly lower for centers when compared to forwards (*p *= 0.048, g = 2.527) and guards (*p *= 0.006, *g *= 1.623). On the other hand, no statistically significant differences were found for concentric impulse/BM (*F*_[2,25] _= 2.947, *p* = 0.071), peak velocity (*F*_[2,25] _= 2.037, *p *= 0.152), mean force (*F*_[2,25] _= 0.833, *p *= 0.446), peak force (*F*_[2,25] _= 0.187, *p *= 0.830), mean power (*F*_[2,25] _= 0.695, *p *= 0.508), and peak power (*F*_[2,25] _= 0.561, *p *= 0.578) force-time metrics.

In addition, no position-specific differences in force-time metrics were detected for CVJ contraction time (*F*_[2,25] _= 0.639, *p *= 0.536), vertical jump height (*F*_[2,25] _= 1.811, *p *= 0.184), and RSI-modified (*F*_[2,25] _= 2.960, *p *= 0.070). See Table 1.

**Table 1:** Definitions of biomechanical parameters examined in the present study.

Variable (unit)	Centers	Forwards	Guards	All players
Eccentric phase				
Braking phase duration (s)	0.313 ± 0.059	0.311 ± 0.083	0.292 ± 0.061	0.301 ± 0.066
Braking impulse (N·s)	78.4 ± 10.2*^,^**	61.3 ± 14.9	58.4 ± 9.3	62.8 ± 13.2
Braking impulse/BM (N·s·kg^−1^)	0.715 ± 0.089	0.624 ± 0.147	0.645 ± 0.101	0.651 ± 0.114
ECC duration (s)	0.505 ± 0.074	0.499 ± 0.103	0.489 ± 0.079	0.494 ± 0.082
ECC peak velocity (m·s^−1^)	−1.37 ± 0.16	−1.27 ± 0.21	−1.24 ± 0.22	−1.27 ± 0.20
ECC mean force (N)	1,078.2 ± 61.7*^,^**	963.6 ± 47.2*	891.7 ± 52.3	945.6 ± 86.7
ECC mean force / BM (N)	9.83 ± 0.02	9.82 ± 0.01	9.83 ± 0.01	9.83 ± 0.01
ECC peak force (N)	2,485.6 ± 129.8	2,377.3 ± 275.3	2,237.5 ± 345.7	2,321.7 ± 306.5
ECC peak force/BM (N)	22.7 ± 0.9	24.2 ± 2.1	24.7 ± 3.5	24.2 ± 2.9
ECC mean power (W)	739.0 ± 48.6*^,^**	602.8 ± 81.8	547.7 ± 90.5	597.6 ± 106.9
ECC mean power/BM (W·kg^−1^)	6.76 ± 0.59	6.12 ± 0.72	6.06 ± 1.07	6.21 ± 0.92
ECC peak power (W)	2,134.4 ± 431.5	1,894.8 ± 683.4	1,677.7 ± 518.6	1,821.3 ± 565.1
ECC peak power/BM (W·kg^−1^)	19.5 ± 4.3	19.2 ± 6.4	18.6 ± 5.9	19.9 ± 5.6
Concentric phase				
CON duration (s)	0.290 ± 0.024*	0.253 ± 0.031	0.241 ± 0.037	0.253 ± 0.037
CON impulse (N·s)	279.4 ± 16.6*	269.9 ± 11.6*	247.4 ± 20.7	259.6 ± 22.0
CON impulse/BM (N·s·kg^−1^)	2.55 ± 0.13	2.75 ± 0.11	2.73 ± 0.19	2.70 ± 0.17
CON peak velocity (m·s^−1^)	2.69 ± 0.13	2.86 ± 0.10	2.84 ± 0.18	2.82 ± 0.16
CON mean force (N)	2,040.0 ± 157.1	2,040.4 ± 198.3	1,938.9 ± 223.6	1,985.9 ± 205.9
CON mean force/BM (N)	18.6 ± 0.5*	20.8 ± 1.4	21.4 ± 2.0	20.7 ± 1.9
CON peak force (N)	2,497.0 ± 150.2	2,467.0 ± 266.7	2,409.1 ± 362.2	2,441.4 ± 301.9
CON peak force/BM (N)	22.8 ± 0.7*	25.1 ± 1.8	26.5 ± 3.0	25.5 ± 3.0
CON mean power (W)	2,908.6 ± 232.1	3,072.3 ± 290.1	2,902.9 ± 386.8	2,952.3 ± 336.7
CON mean power/BM (W·kg^−1^)	26.5 ± 1.4*^,^**	31.3 ± 2.4	32.0 ± 3.8	30.8 ± 3.7
CON peak power (W)	5,094.8 ± 174.0	5,343.8 ± 424.2	5,095.6 ± 551.3	5,166.4 ± 551.3
CON peak power/BM (W·kg^−1^)	46.5 ± 1.9*^,^**	54.5 ± 3.7	56.2 ± 6.7	53.9 ± 6.4
Other				
Contraction time (s)	0.795 ± 0.094	0.752 ± 0.128	0.730 ± 0.107	0.748 ± 0.110
Vertical jump height (m)	0.344 ± 0.004	0.390 ± 0.003	0.387 ± 0.005	0.380 ± 0.005
RSI-modified (m·s^−1^)	0.436 ± 0.045	0.534 ± 0.089	0.541 ± 0.092	0.520 ± 0.091

CON, concentric; ECC, eccentric; RSI-modified, reactive strength index-modified.

* Significantly different when compared to guards.

** Significantly different when compared to forwards (*p* < 0.05).

## 4. Discussion

To the best of our knowledge, this is the first study that examined position-specific differences in force-time metrics within a cohort of professional male basketball players. During the eccentric phase of the CVJ, centers had notably greater braking impulse, mean force, and mean power when compared to guards. While no significant differences were observed between forwards and centers, the magnitude of the eccentric mean force was greater for forwards than guards. However, when normalized by body mass, the observed differences in the aforementioned force-time metrics during the eccentric phase of the CVJ were nonexistent.

Contrary to the findings pertaining to the eccentric phase, no differences in absolute mean and peak force and power were detected between the playing positions during the concentric phase of the CVJ. Interestingly, when expressed relative to the player’s body mass, centers demonstrated inferior performance than guards in the same force-time metrics. Also, concentric mean force and power were greater for forwards than centers, with no differences being observed between forwards and guards. In addition, it should be noted that no position-specific differences were noted in vertical jump height and RSI-modified.

A considerable amount of scientific literature has been focused on analyzing position-specific differences in various performance and physiological parameters in amateur and professional basketball players such as aerobic capacity, anaerobic power, sprinting capabilities, anthropometric characteristics, and isokinetic strength (9, 10, 18–23). Overall, the performance on the majority of these tests (e.g., VO_2max_, 10 and 30 m sprint, peak and mean power during Wingate anaerobic test) seems to be in favor of guards (9, 10, 21). Although focused on examining lower-body neuromuscular performance, our results tend to resemble a similar trend for force-time metrics examined during the concentric phase of the CVJ. When expressed in absolute terms, no significant differences were noted in peak and mean force and power. However, when expressed in relative terms (i.e., adjusted by the player’s body mass), guards demonstrated superior performance in each of the aforementioned variables when compared to centers. Despite being primarily focused on examining the lower-body isokinetic profile of elite male basketball players, Bradic et al. (18) made similar observations where normalization of strength values by body mass canceled out differences in knee extensor and flexor strength. Further, it is interesting to observe that both mean and peak power were notably greater for guards than centers examined in the present study, while no position-specific differences were observed in concentric peak velocity. Thus, we can assume that the greater power outputs observed in guards may be attributed to greater force production capabilities relative to their body mass [i.e., *power = force  *× * **velocity* (24)].

As suggested by previous research reports, the eccentric phase of CVJ should not be overlooked, as a majority of sport-specific movements in basketball require athletes to perform a combination of eccentric and concentric muscle actions (25, 26). It is interesting to note that the apparent positional differences expressed in absolute terms for the majority of force-time metrics examined during the eccentric phase of the CVJ tend to disappear when normalized by the player’s body mass (e.g., braking impulse, mean force and power). This can be largely due to differences in anthropometric characteristics, as centers on both youth and professional levels of basketball competition are taller and heavier than guards and/or forwards (19, 21). Still, rather than assuming that this phase of the CVJ should receive less attention from sports scientists and strength and conditioning practitioners, these findings may actually indicate that all playing positions need to possess similar levels of eccentric strength and power relative to their body mass, especially when taking into account that this group of participants were well-trained and highly-experienced professional athletes.

Lastly, it should be noted that force-time metrics assessed during concentric and eccentric phases of CVJ in professional basketball players examined in the present study were similar in magnitude to a recently published research report studying top-level American football collegiate players (26) as well as unpublished data collected in our laboratory on basketball players competing in first-tier professional leagues in Europe (e.g., ProA League in Germany and France). While these findings can help sports scientists and strength and conditioning practitioners with the design of individually tailored training regimens, further research is warranted to examine the optimal levels of strength and power that these athletes need to possess. Also, future research needs to examine if the findings of the present investigation are gender-specific and if they remain applicable across various levels of basketball competition (e.g., high school, collegiate).

## Data Availability

The raw data supporting the conclusions of this article will be made available by the authors, without undue reservation.
